# Growth and Dark Current Analysis of GaSb- and InP-Based Metamorphic In_0.8_Ga_0.2_As Photodetectors

**DOI:** 10.3390/ma16134538

**Published:** 2023-06-23

**Authors:** Peng Cao, Tiancai Wang, Hongling Peng, Qiandong Zhuang, Wanhua Zheng

**Affiliations:** 1 Laboratory of Solid State Optoelectronics Information Technology, Institute of Semiconductors, Chinese Academy of Sciences, Beijing 100083, China; pengkt11@semi.ac.cn (P.C.);; 2 Center of Materials Science and Optoelectronics Engineering, University of Chinese Academy of Sciences, Beijing 100049, China; 3 College of Electronic and Communication Engineering, University of Chinese Academy of Sciences, Beijing 101408, China; 4 State Key Laboratory on Integrated Optoelectronics, Institute of Semiconductors, Chinese Academy of Sciences, Beijing 100083, China; 5 Physics Department, Lancaster University, Lancaster LA1 4YB, UK

**Keywords:** MBE growth, InGaAs, dark current

## Abstract

Short-wavelength infrared photodetectors based on metamorphic InGaAs grown on GaSb substrates and InP substrates are demonstrated. The devices have a pBn structure that employs an AlGaAsSb thin layer as the electron barrier to suppress dark current density. The strain effect on the electrical performance of the devices was specifically studied through the growth of the pBn structure on different substrates, e.g., InP and GaSb, via a specific buffering technique to optimize material properties and minimize dark current. A lower device dark current density, down to 1 × 10^−2^ A/cm^2^ at room temperature (295 K), was achieved for the devices grown on the GaSb substrate compared to that of the devices on the InP substrate (8.6 × 10^−2^ A/cm^2^). The improved properties of the high-In component InGaAs layer and the AlGaAsSb electron barrier give rise to the low dark current of the photodetector device.

## 1. Introduction

The detection of short-wavelength infrared (SWIR), from 1 μm to 3 μm, has attracted great attention due to a wide range of applications exploited in this spectral band, including fiber-based telecommunication, LiDAR aerospace detection, gas detection, biomedical imaging and identification surveillance [1,2,3,4]. So far, many material systems have been used to fabricate a SWIR photodetector. HgCdTe (MCT) is one of the commercially mature materials covering this spectral band [5,6] due to its widely tunable bandgap. However, poor crystal quality and low uniformity of large material areas still present great challenges to MCT-based devices, which increase the whole cost of the devices both in the fabrication process and final product [7], and typically present difficulty in large area focal plane arrays. In recent years, a newly developed material system, type II superlattice (T2SL) structure, has been exploited to work across the SWIR spectral band range. Photodetectors based on the T2SL system not only obtain the ability to cover wide range spectral band as MCT counterparts, representing great flexibility in band engineering [8,9], but also show advantages in suppressing auger recombination and have a high crystal quality [10,11]. Nevertheless, more complicated growth conditions for abrupt interfaces with suppressed intermixing of As/Sb are required in the case of ultrathin superlattices.

High-indium component (x > 0.53) InGaAs based SWIR photodetectors occupy a favorable position due to their low costs and mature technology in growth [12,13,14]. The concern with InGaAs based SWIR is the lattice mismatch between the high-In component InGaAs absorption layer and the InP substrate. The lattice mismatch rises up to 1.7% for the InGaAs with an In component of 0.78 [15], which leads to great dislocation density during the growth process and a large dark current density. Adequate efforts have been made to reduce the dislocation density and dark current level, including using various buffer layers and growth techniques on different types of substrates. As for the works focusing on buffer layers to reduce the dislocation density in absorption layer, a linearly graded InAlAs buffer [16] and a step-continuously graded InAlAs buffer [17] were reported to moderate the lattice mismatch between high-In component InGaAs and InP substrate. A high crystal quality was obtained due to these buffers, which improved the electrical performance of InGaAs based photodetectors working in the SWIR spectral range. As for the research on improving the growth conditions to improve the crystalline quality of high-In component InGaAs epi-layers, structural and optical properties as well as further growth temperature optimization of GaAs-based In_0.83_Ga_0.17_As materials have been investigated [18].

Another way to further suppress the dark current of high-In component InGaAs photodetector devices is the deployment of electron barriers in different locations of the whole device. Successful barrier choice has been demonstrated in mid-wavelength infrared (MWIR) based on InAs, InAsSb or InAs/Ga(As)Sb superlattice structures, which block minority carriers to suppress Auger combination dark current [19,20] or majority carriers to suppress Shockley–Read–Hall (SRH) dark current [21]. As for the barrier for the SWIR InGaAs based photodetector, InAs/InGaAs superlattice was inserted at different locations in the high-In component InGaAs absorption layer to reduce dark current density [22,23], and an InAlAs layer with the same In component as the InGaAs absorption layer was also exploited [24]. The InAs/InGaAs barrier provides a large conduction band offset (CBO) to block electrons from moving towards the top contact and also presents a small valence band offset (VBO) to guarantee the flow of holes. However, taking advantage of the InAs/InAlAs electron barrier still brings issues of fine control of growth, and exploiting the InAlAs electron barrier presents a small CBO of 153 meV [24].

In this study, we present two pBn type SWIR InGaAs photodetectors with an In component of 0.8 grown on a GaSb substrate and an InP substrate, respectively. A combination of the InAlAs and InAlAs/InGaAs superlattice was exploited as a buffer layer to reduce the dislocation density related to lattice mismatch. Also, an unintentionally doped AlGaAsSb barrier lattice matched to In_0.8_Ga_0.2_As was inserted between the In_0.8_Ga_0.2_As absorption layer and the N-type doped InAlAs top contact layer. Compared with the traditional AlAsSb electron barrier, introducing the Ga element can reduce the possibility of oxidation associated with the high Al component in ternary compounds. Both structural and optical properties of the epitaxy layers grown on the GaSb and InP substrates were presented, and dark current characteristics of photodetectors fabricated on both the GaSb and InP substrates were investigated.

## 2. Experiments

### 2.1. Device Configuration

**Figure 1:**
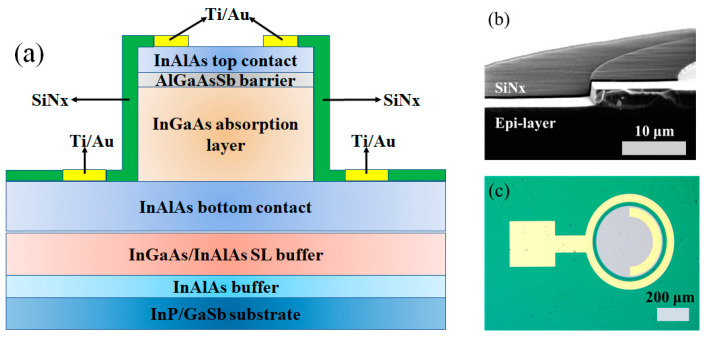
(**a**) Schematic mesa-type pBn In_0.8_Ga_0.2_As photodetector device. (**b**) SEM image of cross-section of SiNx passivated mesa. (**c**) Microscope image of the device.

Figure 1a shows the schematic cross-section of a fabricated mesa-type pBn In_0.8_Ga_0.2_As photodetector. The whole configuration consists of a compound buffer layer of an unintentionally doped 30 nm thick In_0.8_Al_0.2_As and a 600 nm thick In_0.8_Ga_0.2_As/In_0.8_Al_0.2_As superlattice (SL) layer fully relaxing the strain; a 1 μm thick heavily N-type doped In_0.8_Al_0.2_As bottom contact; a 2 μm thick unintentionally doped In_0.8_Ga_0.2_As absorption layer (N-type 1 × 10^16^ cm^−3^); a 50 nm thick unintentionally doped AlGaAsSb barrier (P-type 1 × 10^16^ cm^−3^) and a 200 nm thick heavily P-type doped In_0.8_Al_0.2_As top contact. The whole fabrication was based on a standard photolithography process. The mesa was dry etched by an Inductive Coupled Plasma (ICP) instrument followed by a wet etching process using Buffered Oxide Etchant (BOE) to smooth the mesa surface. After that, 400 nm SiNx was deposited to compensate for the dangling bonds of the mesa side wall. The scanning electron microscope (SEM) image of the SiNx passivated mesa is shown in Figure 1b and the final schematic device under the microscope is shown in Figure 1c.

**Figure 2:**
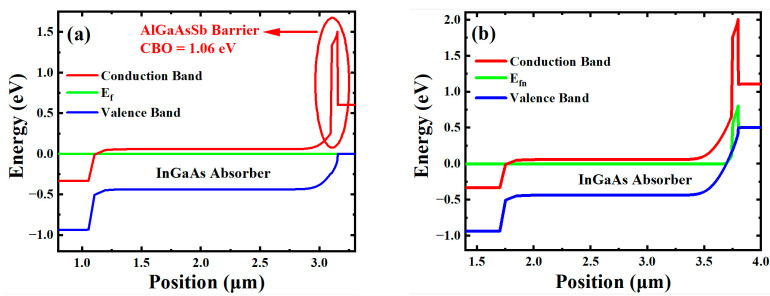
Simulation results of band diagram of pBn In_0.8_Ga_0.2_As photodetector under (**a**) equilibrium condition (**b**) reverse bias of −0.5 V.

Band diagrams of these two pBn In_0.8_Ga_0.2_As photodetectors working under different bias conditions were simulated by SILVACO TCAD 2022 software as shown in Figure 2a,b. Figure 2a shows the band diagram of the device under equilibrium state. According to the band offset between In_0.8_Ga_0.2_As and the AlGaAsSb lattice matched to In_0.8_Ga_0.2_As, a large conduction band offset (CBO) of 1.06 eV and a valence band offset (VBO) of 0.02 eV were represented. Figure 2b shows the band diagram of the device under a reverse bias voltage of −0.5 V. Under reverse bias, the main part of Fermi energy level bending is located in the wide band gap AlGaAsSb barrier, indicating that the applied voltage drops on this barrier layer, which leads to a short depletion region in the absorption layer and thus suppresses the G-R current.

The photodetector structures were grown by molecular beam epitaxy. After the desorption of the native oxides of the substrate, the growth process was started by the deposition of a 30 nm thick In_0.8_Al_0.2_As at a low growth temperature, which was followed by annealing at 450 °C. This was continued with the growth of 30 superlattices of In_0.8_Ga_0.2_As/In_0.8_Al_0.2_As followed by a 1μm thick In_0.8_Al_0.2_As. Then, the pBn structures were grown at pre-optimized growth conditions including a growth rate of 0.8 mL/s, a growth temperature of 450 °C and a V/III beam equivalent pressure ratio of 5–8. High resolution X-ray diffraction (XRD) and atomic force microscopy (AFM) were used to investigate the structural and surface morphology of the grown epitaxy layer structures. Temperature-dependent photoluminescence (PL) was also used to examine the optical properties of the epitaxy layer.

### 2.2. Characterization of Material Quality

**Figure 3:**
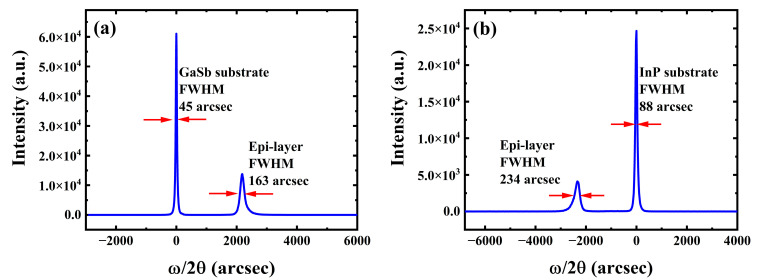
(004) direction ω−2θ X-ray scan of the epitaxy layer grown on (**a**) GaSb substrate and (**b**) InP substrate.

Results of the (004) direction ω-2θ scan for both devices are shown in Figure 3a,b. Both devices present two main peaks in the XRD scan. In Figure 3a, the narrower peak with a full width at half maximum (FWHM) of 45 arcsec and the broader peak with a FWHM of 163 arcsec point to the GaSb substrate and the In_0.8_Ga_0.2_As epitaxial absorption layer, respectively. In Figure 3b, the narrower peak with a FWHM of 88 arcsec corresponds to the InP substrate and the broader peak with a FWHM of 234 arcsec corresponds to the high-In component In_0.8_Ga_0.2_As epitaxial absorption layer. Since the lattice constant of the In_0.8_Al_0.2_As buffer and top contact is almost the same as that of In_0.8_Ga_0.2_As, the peak of In_0.8_Al_0.2_As nearly coincides with the broader In_0.8_Ga_0.2_As peak [14]. Also, the peak of the AlGaAsSb barrier lattice matched to In_0.8_Ga_0.2_As is merged in the broader peak for the In_0.8_Ga_0.2_As epitaxial absorption layer in both substrate cases.

**Figure 4:**
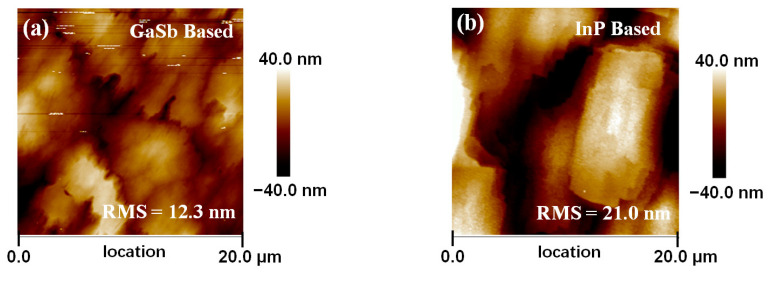
AFM image of In_0.8_Ga_0.2_As surface grown on (**a**) GaSb and (**b**) InP substrate.

A typical AFM image with 20 × 20 μm^2^ scan area is shown in Figure 4a,b. Root-mean-square (RMS) roughness extracted from the AFM images indicates the quality of the surface morphology after the growth of the epitaxy layer on the substrate. The RMS roughness of these In_0.8_Ga_0.2_As surfaces are 12.3 nm and 21 nm, for the structures grown on GaSb and InP, respectively. This indicates that there are fewer dislocations formed when the thickness of the lattice mismatch layer is beyond the critical thickness during the growth on the GaSb substrate. The smoother the surface of InGaAs on GaSb, the better crystalline quality of InGaAs on GaSb in comparison with that of InP, which is correlated with the X-ray diffraction results. We believe that this is associated with the different type of strain in the epilayer during the growth, i.e., a tensile strain grown on a GaSb substrate (lattice mismatch of −2.1%) and a compressive strain grown on an InP substrate (lattice mismatch of +1.9%) [25].

**Figure 5:**
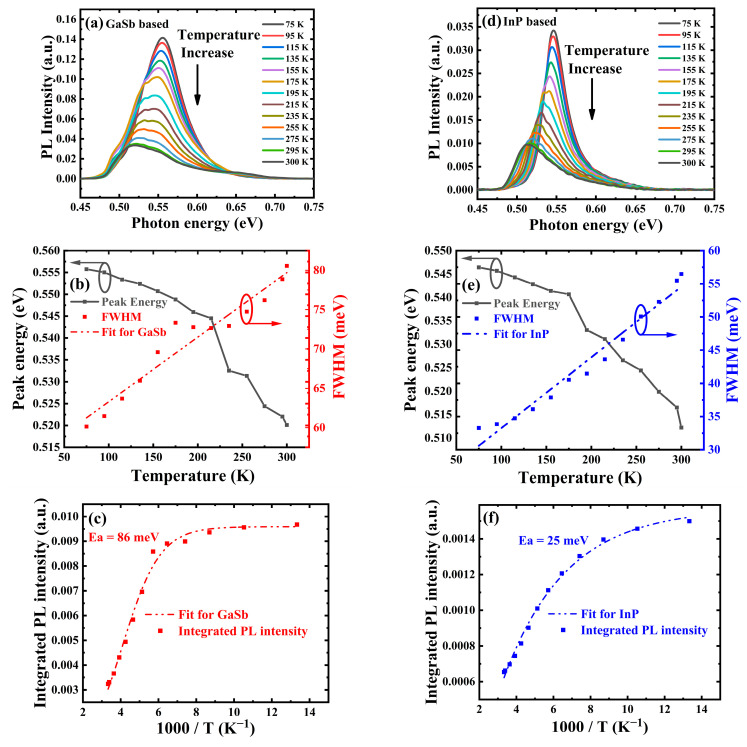
Temperature dependent PL spectra of In_0.8_Ga_0.2_As grown on (**a**) GaSb and (**d**) InP substrate. PL peak energy (grey solid line) and PL FWHM (dash-dotted line) of In_0.8_Ga_0.2_As grown on (**b**) GaSb and (**e**) InP substrate vary as temperature. Temperature dependent integrated PL intensity of In_0.8_Ga_0.2_As grown on (**c**) GaSb and (**f**) InP substrate.

The PL spectra and related analyses for both pBn structures are presented in Figure 5a–f. It can be seen that in both cases, the PL intensity decreases significantly with increasing temperature from 75 K to 300 K, which is attributed to the increased non-radiative recombination. A significant decrease in PL energy is also visible, which coincides with the decreasing InGaAs bandgap as temperature rises, and is also attributed to excitons delocalizing to a high energy level when the thermal activation energy exceeds 75 K [12]. It is also shown in Figure 5a,d that under the same temperature, the PL intensity of the GaSb substrate case is more than three times larger than that of its InP substrate counterpart, which reveals reduced non-radiative recombination centers [17] and improved crystalline quality of the epitaxy layer grown on GaSb. In Figure 5b,e, the PL peak moves from 0.56 eV (2.23 μm) to 0.52 eV (2.38 μm) in the GaSb case and moves from 0.55 eV (2.27 μm) to 0.51 eV (2.42 μm) in the InP case while temperature rises from 77 K to 300 K. Furthermore, it shows a homogeneous temperature-dependence of FWHM of the PL spectra as a result of phonon and exciton scattering, which is shown in Figure 5b,e. Linear fits for the temperature-dependent PL FWHM show a slope of 0.08 meV/K and 0.10 meV/K for epilayers grown on the GaSb and InP substrates, respectively. The linear relationship between the FWHM and temperature indicates that the recombination process in both samples is like a band-to-band type transition [26,27]. Figure 5c,f present the temperature dependent integrated PL intensities of both epilayers grown on the GaSb and InP substrates. Thermal activation energies of 86 meV and 25 meV are extracted for the GaSb and InP cases, respectively. The higher activation energy can be associated with a lower density of threading dislocation generated during the growth of the metamorphic epilayer on GaSb compared with that grown on InP. This could be due to the relaxation mechanism in the tensile and compressive strains. Our results reveal that the tensile strain may lead to less creation of threading dislocation density. This explanation is further supported by the dark current study.

## 3. Dark Current Analysis of Photodetectors

**Figure 6:**
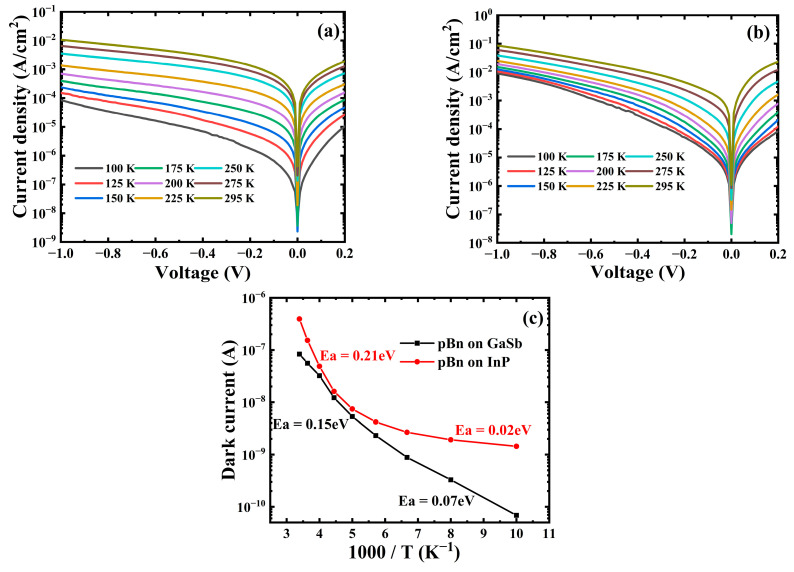
Dark current characteristic of pBn In_0.8_Ga_0.2_As device grown on (**a**) GaSb substrate and (**b**) InP substrate at different temperatures. (**c**) Arrhenius plot of the two devices at reverse bias of −10 mV.

Figure 6a,b shows the measured dark current–voltage characteristics of a pBn type In_0.8_Ga_0.2_As device grown on InP and GaSb at various temperatures. For pBn on the GaSb substrate case, the dark current density is 1 × 10^−2^ A/cm^2^ at room temperature (295 K) and 8.5 × 10^−5^ A/cm^2^ at 100 K, respectively, under a reverse bias of −1 V. For pBn on the InP substrate case, under a reverse bias of −1 V, the dark current density is 8.6 × 10^−2^ A/cm^2^ at room temperature (295 K) and 9 × 10^−3^ A/cm^2^ at 100 K. An Arrhenius plot under a reverse bias of −10 mV is shown in Figure 6c. In the temperature range above 200 K, activation energies of 0.15 eV and 0.21 eV are extracted for devices grown on the GaSb and InP substrate, respectively. For the InP substrate case, this extracted activation energy is nearly half of the bandgap of the In_0.8_Ga_0.2_As layer (0.48 eV), which indicates that the Shockley–Read–Hall (SRH) related G-R current still dominates in this temperature range. While in the GaSb substrate case, the extracted energy above 200 K is lower than half of the value of the bandgap of the In_0.8_Ga_0.2_As layer, which shows that dark current mechanisms other than G-R current still exist in this temperature region. When the temperature drops below 200 K, the extracted activation energies of GaSb and InP substrate are 0.07 eV and 0.02 eV, respectively, which are both much smaller than the bandgap of the In_0.8_Ga_0.2_As layer. This phenomenon indicates that trap-assisted tunneling currents caused by a large amount of dislocations dominates below 200 K [28]. In this temperature range, a larger extracted activation energy Ea from the device on the GaSb substrate means trap-assisted tunneling current related to dislocations and surface leakage is lower, while in a higher temperature range these effects still contribute. Table 1 shows the dark current comparison under a reverse bias of −1 V of the high-In component InGaAs photodetector based on different substrates. The GaSb based high-In component InGaAs photodetector in this work still shows advantages over its GaAs based counterparts, which presents the potential for further research of high-In component InGaAs epilayers grown on different substrates. The optimization of MBE growth conditions to reduce dislocations is needed to further lower this trap-assisted tunneling current, and improvement of the electron barrier is required to suppress G-R related dark current.

## 4. Conclusions

In conclusion, we have investigated the structural and optical properties of metamorphic In_0.8_Ga_0.2_As grown on GaSb and InP substrates, and the dark current characteristics of a pBn In_0.8_Ga_0.2_As device with an AlGaAsSb barrier inserted, fabricated on both GaSb and InP substrates. Atomic force micrographs and PL spectra intensity show a smoother surface and improved optical property of the In_0.8_Ga_0.2_As epilayer grown on a GaSb substrate. At 295 K, the dark current densities of the pBn In_0.8_Ga_0.2_As devices grown on the GaSb and InP substrates are 1 × 10^−2^ A/cm^2^ and 8.6 × 10^−2^ A/cm^2^, respectively. At 100 K, the dark current densities of device grown on the GaSb and InP substrates are 8.5 × 10^−5^ A/cm^2^ and 9 × 10^−3^ A/cm^2^, respectively. Trap-assisted tunneling current related to dislocations dominates below 200 K for both devices grown on the GaSb and InP substrates, and the device grown on GaSb has a lower dark current than the device grown on the InP substate, which attributed to the improved structural and optical properties of the high-In component InGaAs absorption layer.

## Figures and Tables

**Table 1:** Dark current comparison at reverse bias −1 V.

	[18]	[28]	This Work
Substrate	GaAs	GaAs	GaSb
Dark current density	0.15 A/cm^2^	0.08 A/cm^2^	0.01 A/cm^2^

## Data Availability

The data that support the findings of this study are available from the corresponding author upon reasonable request.
